# Type 2 autoimmune pancreatitis associated with ulcerative colitis

**DOI:** 10.3389/fimmu.2023.1288390

**Published:** 2023-12-06

**Authors:** Nan Nan, Dongxu Wang

**Affiliations:** Department of Gastroenterology, Shengjing Hospital of China Medical University, Shenyang, China

**Keywords:** ulcerative colitis, autoimmune pancreatitis, inflammatory bowel disease, prevalence, pathogenesis, treatment

## Abstract

Ulcerative colitis (UC) is a chronic idiopathic inflammatory disease mainly affecting the rectum and colon and causing diarrhoea and mucopurulent stools. UC can present with extraintestinal manifestations in various organs and systems and can be associated with various comorbidities. Autoimmune pancreatitis (AIP) is a specific type of pancreatitis associated with autoimmune abnormalities and is divided into two clinical types: type 1 (lymphoplasmacytic sclerosing pancreatitis) and type 2 (idiopathic ductocentric pancreatitis). The current study shows an association between type 2 AIP and UC, which may be related to genetic susceptibility, inflammatory factors, and immune response. The most common manifestation of AIP in patients with type 2 AIP–UC is abdominal pain with elevated pancreatic enzymes, whereas the presentation of UC in type 2 AIP–UC is more severe, with an increased risk of UC-related surgery. This review focuses on diagnosis, prevalence, pathogenesis, impact, and treatment to better understand type 2 AIP–UC, explore the molecular mechanisms of this condition, and encourage further research into the management of type 2 AIP–UC.

## 1 Introduction

Ulcerative colitis (UC), a major clinical subtype of inflammatory bowel disease (IBD), is a chronic idiopathic intestinal inflammatory disease (1). UC primarily involves the rectum, colonic mucosa, and submucosa (2), with abdominal pain, diarrhoea, and mucopurulent bloody stools as its main manifestations (3). Patients with UC may develop varying degrees of extraintestinal manifestations, including skin, joint, ocular, hepatic, and pulmonary disorders (4), and may have multiple comorbid conditions. The incidence and prevalence of UC are increasing with the westernisation of newly industrialised countries, and the incidence of IBD in children worldwide is rising, making UC a global disease (5, 6). The disease is currently unpredictable, recurrent and requires long-term treatment.

Autoimmune pancreatitis (AIP) was originally proposed in 1995 by Yoshida et al. to describe a group of pancreatitis cases associated with autoimmune abnormalities, resulting in hypergammaglobulinaemia or autoantibody positivity, that impair the effectiveness of glucocorticoid (GC) therapy (7). The clinical signs and symptoms of AIP vary and are nonspecific, often presenting as acute pancreatitis (AP), abdominal pain, and obstructive jaundice (8, 9). AIP presents with diffuse or focal enlargement of the pancreas and has two types of pathology: lymphoplasmacytic sclerosing pancreatitis (LPSP) and idiopathic duct-centric pancreatitis (IDCP) (10, 11). In 2010, the International Pancreatic Society classified AIP into two clinical types, type 1 AIP (corresponding to LPSP) and type 2 AIP (corresponding to IDCP), and established international consensus diagnostic criteria (ICDC) (12). These two types have similar imaging presentations but different clinical features (13). Type 1 AIP is a pancreatic manifestation of systemic immunoglobulin G4-related disease (IgG4-RD) (14, 15). Elevated serum IgG4 levels and evidence of the involvement of other organs are of high diagnostic value for type I AIP; therefore, histopathology is not necessary for the diagnosis of type 1 AIP. Type 2 AIP is currently not considered a systemic disease and mainly involves the pancreas. The lack of specific biomarkers and low positivity for serum IgG4 and other autoantibodies makes its diagnosis extremely challenging.

Although the aetiology and pathogenesis of UC and type 2 AIP are unclear, some studies have demonstrated an association between these diseases. AIP is currently considered as an autoimmune disease that can coexist with IBD; however, whether type 2 AIP is an extraintestinal manifestation of UC is controversial, as it is unclear whether its pathogenesis is related to the intestinal immune response (16, 17). The current study shows that most patients with AIP complicated by IBD have type 2 AIP, and its association with UC is stronger than that with Crohn’s disease (CD) (18). Understanding the clinical relevance and potential pathogenesis of type 2 AIP–UC may help to formulate reasonable treatment strategies. Patients with IBD presenting with abnormal pancreatic enlargement are diagnosed with probable type 2 AIP without histology if the pancreatic abnormalities resolve or improve rapidly after GC treatment, after excluding any associated malignancy. Meta-analyses have shown that patients with UC are at an increased risk of pancreatitis compared with the non-IBD population (19, 20). Pharmacological pancreatitis can be caused by drugs such as azathioprine, so in these situations, this treatment should be discontinued; however, in the case of AP manifestations due to concurrent type 2 AIP, unnecessary discontinuation should be avoided (21).

Knowledge of type 2 AIP has gradually increased in recent years, but studies on type 2 AIP–UC remain relatively scarce. Therefore, we reviewed from the diagnosis, prevalence, possible mechanisms, impact, and treatment and challenge of type 2 AIP–UC to guide the management of type 2 AIP–UC in clinical practice.

## 2 Diagnosis of type 2 AIP–UC

**Figure 1:**
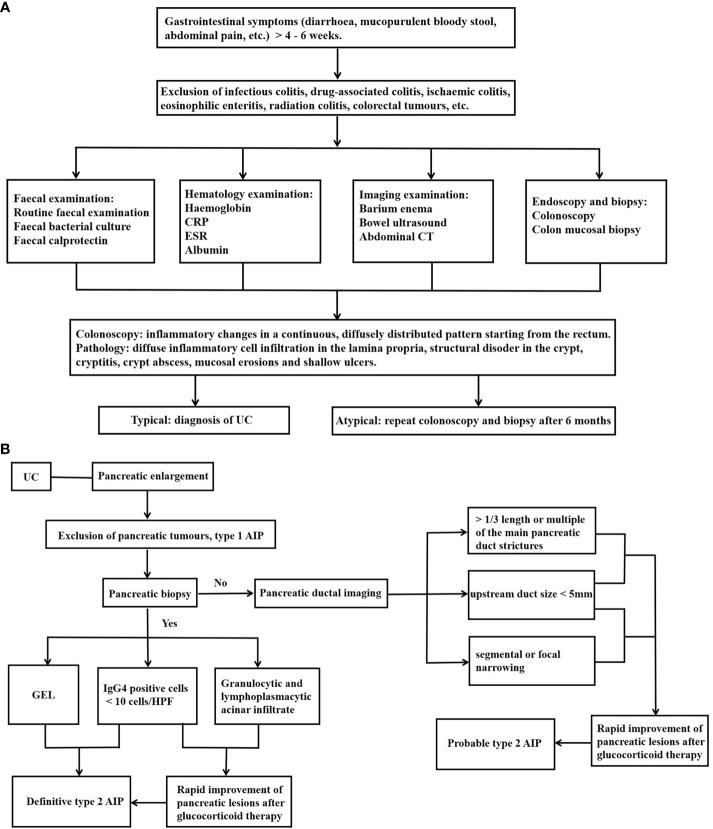
Diagnosis of Type 2 AIP–UC. **(A)** Diagnosis of UC; **(B)** Diagnosis of Type 2 AIP–UC. UC, ulcerative colitis; CRP, C-reactive protein; ESR,erythrocyte sedimentation rate; CT, computed tomography; GEL, granulocyte epithelial lesions; AIP, autoimmune pancreatitis; IgG, immunoglobulin G; HPF, high-power field.

The diagnosis of UC is based on the exclusion of infections, drugs, radiotherapy, ischaemic enteritis and colorectal tumours, and requires the judgement of a specialist IBD practitioner based on a combination of gastrointestinal symptoms, colonoscopy, pathology and biochemical indices, as detailed in **Figure 1A** (22, 23). Faecal calreticulin (FC) is strongly correlated with the degree of endoscopic inflammation and is valuable in the assessment of initial diagnosis, recurrence and response to treatment (24).

Most patients with type 2 AIP–UC were diagnosed with UC first or both at the same time (25–29). Diagnostic criteria for AIP include the Japanese JPS criteria (30), the Korean Kim criteria (31), the American HISORt criteria (32), and the Asian criteria (33). However, none of them differentiate between AIP types and focus on describing the characteristics of type 1 AIP. Consequently, non-IgG4 related type 2 AIP may not be well represented. Although the Italian Verona standard (34), developed in 2009, contains the characteristics of both types of AIP, it does not distinguish between them. ICDC is currently considered to have the highest accuracy and sensitivity for the diagnosis of AIP and is the most useful for the classification of type 1 and type 2 AIP (35). A recent large international cohort study of AIP–IBD has shown that ICDC is the most frequently used diagnostic criterion for AIP (29).

The ICDC-based diagnostic process for autoimmune pancreatitis is summarised in **Figure 1B** (12, 29). As can be seen, the presence of UC is one of the criteria for the diagnosis of probable type 2 AIP, so patients with a clear diagnosis of UC may still be diagnosed with probable type 2 AIP in the absence of a pancreatic biopsy. Bowel-related investigations in AIP patients with equivocal histopathology are also very important, and we can routinely assess the FC level at the time of diagnosis and during the follow-up period, and in the event of an FC elevation, even in the absence of intestinal symptoms, colonoscopy should be refined for early recognition of UC (26). Although histological examination is not mandatory for the diagnosis of probable type 2 AIP, pancreatic biopsy remains important for type 2 AIP. A retrospective study found that the proportion of patients with pancreatitis who underwent histological examination at the centre increased from 1% to 3% as time progressed, and that the proportion of patients with type 2 AIP to total AIP increased from 8% to 55% (36). In addition, the differentiation between type 2 AIP, which presents as a mass pattern, and pancreatic cancer is challenging (37). Studies have reported that 78% of patients with type 2 AIP who obtained a histological diagnosis of the pancreas underwent surgical resection on suspicion of pancreatic cancer (38). Enhanced magnetic resonance, PET-CT, ultrasonographic endoscopy, and serum CA199 may be helpful in this distinction (39).

Serological markers also present some value in the diagnosis of type 2 AIP–UC. Anti-neutrophil cytoplasmic antibodies (ANCA) are mainly divided into perinuclear ANCA (p-ANCA) and cytoplasmic ANCA (c-ANCA) (40). Serum p-ANCA and anti-saccharomyces cerevisiae antibodies have high specificity but low sensitivity in differentiating UC from CD (41–43). Serum Proteinase 3 ANCA (44, 45) and IgG anti-integrin αvβ6 autoantibodies (46, 47) may be potential diagnostic markers for UC. However, serological markers specific for type 2 AIP have not yet been clearly identified (48), and only a few studies that included a small number of samples have conducted preliminary explorations. Serum levels of anti-transaldolase antibodies were significantly higher in type 2 AIP than in type 1 AIP and pancreatic ductal adenocarcinoma (49). Combination of serum IgG4 and anti-amylase-α antibodies may help to diagnose and differentiate between AIP subtypes and rule out pancreatic cancer (50). In addition, neither p-ANCA nor c-ANCA were detected to be elevated in type I AIP, whereas they were elevated in 50% and 30% of type 2 AIP patients (51, 52). Anti-smooth muscle antibodies were elevated in 50% of type 2 AIP patients, compared with 17% of type 1 AIP patients (51). These findings on serological markers of type 2 AIP still need to be validated by larger studies.

## 3 Prevalence of type 2 AIP–UC

The association between AIP and IBD is well established, particularly between UC and type 2 AIP (52–55). The prevalence of IBD is significantly higher in patients with AIP (approximately 11%–30%) (54, 56, 57). Approximately 30%–48% of type 2 AIP cases are associated with IBD (9, 58, 59). Most patients with AIP and comorbid IBD have type 2 AIP (9). A national survey of patients with IBD–AIP in Japan showed that UC accounts for 73% of patients with concurrent type 2 AIP and IBD (type 2 AIP–IBD), which is significantly higher than the incidence of CD (60).

### 3.1 Prevalence of UC in patients with type 2 AIP

The prevalence of UC in type 2 AIP reported in recent studies ranges from 16%–83% (25–27, 38, 57, 59, 61–68), as shown in **Table 1**. Among these, the studies by Detlefsen et al. (62) and Kamisawa et al. (38) were in the context of histologically diagnosed type 2 AIP, which may have reduced the inclusion of patients with actual type 2 AIP. The studies by Notohara et al. (64), Park SH et al. (65), Schneider A et al. (57) and Czakó et al. (67) included a small number of patients with type 2 AIP, and the results may not adequately reflect the actual situation. We found that both studies from Italy used the ICDC diagnosis, but the results differed significantly for the following possible reasons: Barresi et al.’s study (62) was a national survey that included 173 centres and the diagnosis of AIP was based on the independent judgement of each centre, whereas Conti et al.’s study (26) was a single-centre study, so the heterogeneity of diagnosis was different between the two studies. In addition, about three times as many cases of type 2 AIP were histologically examined in the study of Barresi et al. (62) than that of Conti et al. (26), and the majority of type 2 AIP cases in Conti et al.’s study were probable diagnoses (26), which may have contributed to the high prevalence of type 2 AIP.

**Table 1:** Prevalence of UC in patients with type 2 AIP.

Author	Year	Region	Diagnostic Criteria for AIP	Size		Prevalence
				Type 2 AIP	UC	
Czakó L (67)	2011	Hungary	ICDC	3	2	67%
Kamisawa T (38)	2011	Japan, Korea, India, USA, Germany	ICDC	64	10	16%
Detlefsen S (63)	2012	Denmark	ICDC	51	8	16%
Park SH (65)	2013	Denmark	ICDC	5	4	80%
Notohara K (64)	2015	Denmark	ICDC	8	2	25%
Hart P.A (27).	2016	USA	ICDC	43	14	33%
Ku Y (68)	2017	Japan	ICDC	13	5	38%
Buechter M (61)	2017	Germany	ICDC	16	3	19%
Schneider A (57)	2018	Germany	ICDC	7	3	43%
Oh D (25)	2019	Germany	ICDC	27	12	44%
Barresi L (62)	2020	Italy	ICDC	48	11	23%
Conti Bellocchi MC (26)	2022	Italy	ICDC	54	45	83%
Goni E (66)	2022	Italy, Germany	ICDC	23	11	48%
Nikolic S (59)	2022	Sweden, Italy	ICDC	35	17	59%

AIP, autoimmune pancreatitis; ICDC, international consensus diagnostic criteria; UC, ulcerative colitis.

### 3.2 Prevalence of type 2 AIP in UC patients

Few studies have explored the prevalence of type 2 AIP in UC, with only two large-sample studies with reliable data from Japan and Korea (16, 69), as detailed in **Table 2**.

**Table 2:** Prevalence of type 2 AIP in patients with UC.

Author	Year	Region	Diagnostic Criteria for AIP	Size		Prevalence
				UC	Type 2 AIP	
Ueki T (16)	2015	Japan	ICDC	961	5	0.5%
Kim JW (69)	2017	Korea	ICDC	3302	13	0.4%

AIP, autoimmune pancreatitis; ICDC, international consensus diagnostic criteria; UC, ulcerative colitis.

## 4 Possible pathogenesis of type 2 AIP–UC

**Figure 2:**
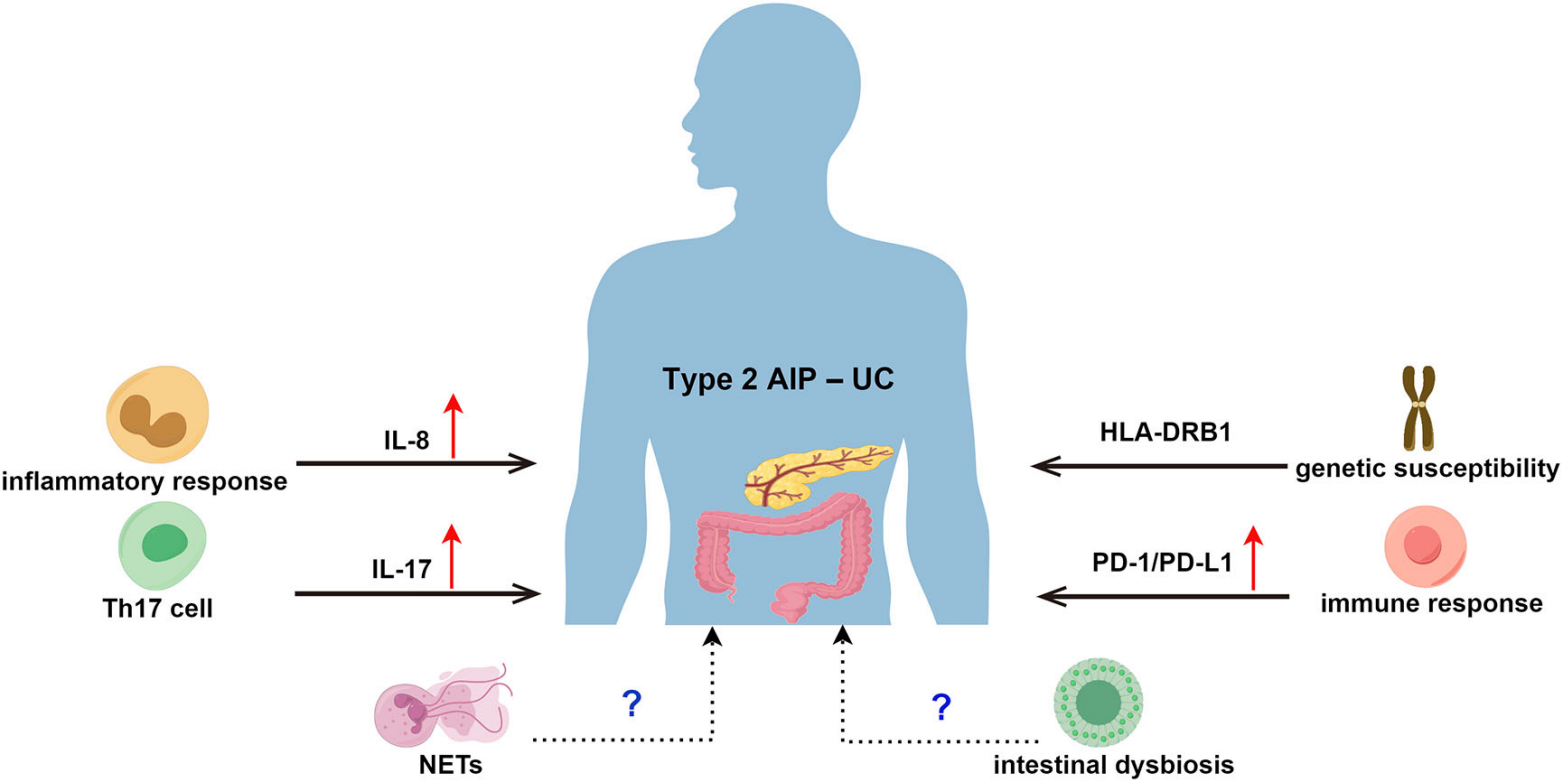
Possible Pathogenesis of Type 2 AIP–UC. AIP, autoimmune pancreatitis; UC, ulcerative colitis; HLA, human leukocyte antigen; IL-8, Interleukin 8; Th 17, T helper 17; IL-17, Interleukin 17; PD-1, programmed death receptor 1; PD-L1, programmed cell death ligand 1; NETs, neutrophil extracellular traps.

The pathogenesis of type 2 AIP is not fully understood, but its clinical relevance in UC suggests that there may be a common pathogenic mechanism (70, 71). From a histopathological point of view, there is a large intraepithelial neutrophil infiltration in both the granulocyte epithelial lesions (GEL) of type 2 AIP and cryptitis and crypt abscesses in UC (72, 73), which suggests a possible common pathogenesis (74). Patients with concurrent active UC and type 2 AIP have been found to experience concurrent relief after anti-tumour necrosis factor (TNF) treatment, suggesting that the two conditions may share similar pathophysiological mechanisms (75). The pathogenesis of type 2 AIP–UC may be related to genetic susceptibility, interleukin (IL)-8, T helper (Th) 17 cells and related cytokines, and programmed cell death ligand 1 (PD-L1), as shown in **Figure 2**.

### 4.1 Genetic susceptibility

The human leukocyte antigen (HLA) is closely associated with immune tolerance. HLA molecules contain antigenic peptides that are recognised by T-cell receptors, resulting in an immune response. HLA is highly polymorphic (76), and allelic variants can alter its interaction with antigenic peptides and T cell receptors, thereby affecting the immune response (77). Specific HLA allelic variants, particularly HLA class II molecules, are strongly associated with the development of autoimmune diseases (AD), particularly HLA class II molecules (78–81). Destructive inflammatory responses to autoantigens in the context of genetic susceptibility have been suggested as potential pathogenic mechanisms of UC (82). Studies have shown that the HLA gene that is most associated with UC and CD is HLA-DRB1, with HLA-DRB1*03:01 being the predominant risk allele (83, 84). In 2002, Kawa et al. first reported that specific HLA alleles might be associated with increased susceptibility to AIP (85). Goni et al. prospectively investigated HLA alleles in 100 AIPs from Italy and Germany and reported that, despite the different histopathological characteristics of type 1 and type 2 AIPs, both subtypes were associated with a higher frequency of HLA-DRB1*16 alleles and a significant enrichment of HLA-DQB1 pure congeners (especially the HLA-DQB1*05 allele) compared to healthy controls, hypothesising that type 1 and type 2 AIPs share the same genetic susceptibility and may rely on similar immunogenic mechanisms (66). The role of HLA in the pathogenesis of type 2 AIP and UC remains unclear because of the high degree of linkage disequilibrium between HLA genotypes. However, the exact genetic risk loci and functional validation of HLA variants have not been fully elucidated, and further studies are needed to determine whether enrichment of specific HLA haplotypes leads to increased numbers of self-reactive T cells or autoantibody production (85–87).

### 4.2 Interleukin 8

IL-8 is a chemokine that is produced by a variety of cell types involved in inflammation, including monocytes, which can recruit a variety of immune cells, such as neutrophils and T lymphocytes, to the site of inflammation, leading to a range of responses, including inflammatory damage and tissue infiltration (88). In 2007, Ku et al. first examined the immunological profile of type 2 AIP lesions and revealed the presence of overexpressed IL-8 in ductal epithelial cells and infiltrative inflammatory cells; they found a similar pattern of IL-8 expression in crypt epithelial cells from colonic biopsy samples of patients with active UC (68). Similarly, Pearl et al. found that IL-8 levels in the diseased mucosa of patients with UC were positively correlated with the grade of inflammation and that IL-8-mediated infiltration of neutrophils may be associated with the inflammatory response in UC (89). IL8 may be the causative mechanism of type 2 AIP–UC.

### 4.3 Th17 cells and related cytokines

Th17 cells are a subpopulation of CD4+ Th cells that secrete cytokines such as IL-17A, IL-17F, and IL-22 (90), which have been shown to be associated with tissue inflammation and play an important role in the pathogenesis of many forms of AD (91, 92). An imbalance between regulatory and effector T cells (e.g., Th1, Th2, and Th17) is one of the key mechanisms in the immunopathology of UC, and the overactivation of Th17 cells may contribute to the development of UC (93). Patients with active UC have significantly higher numbers of Th17 cells in intestinal tissues and peripheral blood and significantly higher expression of related cytokines, such as IL-17 (94–96). Loos et al. found that Th17 cells infiltrate more significantly around the ducts in the pancreatic tissue in type 2 AIP than in type 1 AIP, accompanied by a significant increase in the number of neutrophils and a significant increase in IL-17A expression (97). Th17 cells and related cytokines are possibly involved in the mechanisms for the coexistence of UC and type 2 AIP.

### 4.4 Programmed cell death ligand 1

Programmed death receptor 1 (PD-1) is a co-receptor expressed on lymphocytes, monocytes, and natural killer cells and has two ligands, PD-L1 and PD-L2 (98). The PD-1/PD-L1 pathway may be involved in lymphocyte activation, T-cell development and function, immune tolerance breakdown, and AD development (99). An increasing number of studies have reported the upregulation of PD-L1 expression in the colonic epithelial cells of patients with UC, suggesting that PD-L1 may be involved in intestinal mucosal immune regulation (100–102). Gupta et al. found that 69% of samples from pancreatic ducts with type 2 AIP showed PD-L1 positive immunoreactivity, suggesting that pancreatic ductal PD-L1 may be associated with abnormal immune responses in type 2 AIP; they determined the sensitivity and specificity of PD-L1 as a marker for type 2 AIP were 70% and 99%, respectively (103). PD-L1 may also be a potential therapeutic target for the type 2 AIP–UC.

### 4.5 Other possible mechanisms to be investigated

Neutrophil extracellular traps (NETs) are released from activated neutrophils, and their structure consists of a meshwork of desmosomal chromatin fibres decorated with antimicrobial granule proteins, such as myeloperoxidase (MPO), neutrophil elastase, histone, and prothrombin G (104). NETs can act as traps that immobilise and kill microorganisms, thus limiting their spread (105). However, if NETs are overproduced and persistent, they can lead to local tissue destruction, a vicious cycle of inflammatory response, and overactivation of immune cells (106). NETs are associated with a variety of immune disorders. Dinallo et al. showed that excessive formation of NETs was present in UC, predominantly in mucosal areas with active inflammation, and that inhibition of NETs formation improved dextran sulphate sodium-induced colitis (107). Several studies have noted that pancreatic tissue from IgG4-related AIP, which is type 1 AIP, contains NETs (14, 108, 109), and NETs-related proteins are overexpressed in the inflamed colon of UC patients (107). Although there are no reports of NETs associated with type 2 AIP, the histological feature of type 2 AIP is granulocytic epitheliopathy (110), so it is reasonable to speculate that NETs might play a role in type 2 AIP–UC. In addition, alterations in the composition and function of the intestinal microbiome (also known as intestinal dysbiosis) have been associated with digestive disorders (111, 112), and the role of intestinal dysbiosis in the pathogenesis of ulcerative colitis has been widely recognised (113, 114). Recent studies have found that intestinal dysbiosis is also closely related to pancreatic diseases, and that intestinal dysbiosis may exacerbate chronic inflammation in type 1 AIP through activation of plasmacytoid dendritic cells producing IFN-α and IL-33, and TLR7-expressing M2 macrophages (115). Although there are no reports of intestinal dysbiosis associated with type 2 AIP, it is hypothesised that excessive innate immune responses of the intestinal dysbiosis may be associated with type 2 AIP–UC.

## 5 Clinical features of type 2 AIP–UC

### 5.1 Clinical manifestations of type 2 AIP–UC

Type 2 AIP mostly occurs after or in conjunction with the diagnosis of UC (25, 26, 28). A national survey in Japan found that abdominal pain was more common in patients with type 2 AIP–IBD than type 1 AIP, whereas the incidence of lower bile duct stricture and obstructive jaundice was significantly lower than in type 1 AIP (60). European studies have shown that the most common presentation of AIP in patients with type 2 AIP–UC is AP (56%–83%), i.e., abdominal pain with elevated pancreatic enzymes, but symptoms are generally mild, without local or systemic complications, and the response to GC is rapid, with persistent pain generally subsiding rapidly after the initiation of GC (26, 28). The Montreal typing of UC in patients with type 2 AIP–UC is predominantly left hemicolonic (E2) and extensively colonic (E3), with the rectal type (E1) being less common (9, 16, 25, 26, 28). Approximately 50%–70% of patients have UC in the active phase of the disease at the time of diagnosis of type 2 AIP (16, 26). Park et al. found that patients with AIP–UC had a lower body mass and higher CRP and Mayo scores than patients with UC without AIP, suggesting an increased severity of UC in patients with AIP–UC (65). Patients with type 2 AIP–UC have nearly twice the rate of severe UC compared to those with UC not complicated by type 2 AIP (16).

### 5.2 Recurrence rate of type 2 AIP

The recurrence of type 2 AIP usually appears on abnormal pancreatic imaging, with or without associated clinical manifestations such as abdominal pain and acute pancreatitis (52, 58). Recurrence rate of type 2 AIP is approximately 5%–34% (9, 25, 26, 38, 59, 116). Ueki et al. continued to follow up patients with UC for approximately 4 years after the diagnosis of type 2 AIP and found that 20% of patients developed type 2 AIP recurrence (16). In an Italian study, a recurrence rate of 11.1% was observed in patients with type 2 AIP–UC at a median follow-up of approximately 4–5 years, and the risk of recurrence was not higher in patients with type 2 AIP–UC compared with those without concurrent UC (26).

### 5.3 Surgical risks of UC

A multicentre retrospective study found that the proportion of patients with type 2 AIP–UC who underwent colectomy was approximately 20%, which was significantly higher than that of patients with UC without concurrent type 2 AIP (5%), and surgery was mostly performed after a diagnosis of type 2 AIP; the majority of UC patients requiring surgery were type E3, and the reason for surgery was overwhelmingly due to the development of acute severe colitis (9). According to the Mayo Clinic, 43% of patients with type 2 AIP–UC undergo colectomy for refractory UC (27). However, different views exist regarding the rate of surgery for UC in patients with type 2 AIP–UC. Bellocchi et al. followed patients for a mean of 55 months and found a colon resection rate of 4%–5% in patients with type 2 AIP–UC, with no increase in colon resection rates compared with patients with UC without concurrent type 2 AIP (26).

### 5.4 Long-term complications

A multicentre retrospective study in Italy reported that 31% of patients with type 2 AIP had pancreatic atrophy, 8% had diabetes mellitus (DM), and 10% had pancreatic calcification after 2–3 years of follow-up (62). After 4–5 years of follow-up of patients with type 2 AIP–IBD, it was found that approximately 19%–28% of patients had pancreatic exocrine insufficiency, and 12%–17% of patients had DM (9, 59). The current study showed that none of the patients with type 2 AIP-UC developed pancreatic or colorectal cancers during the follow-up period (9, 27, 59).

## 6 Treatment and challenges of Type 2 AIP–UC

The preferred treatment option for most type 2 AIP–UC patients is GC. The role of GC in the treatment of UC has been well documented over decades of clinical experience (117), and type 2 AIP also responds well to GC therapy (39). GC inhibit Th-1 and Th-17 differentiation, promote Th2 differentiation and Treg production, induce immunosuppression, and attenuate inflammatory responses (118). TNF inhibitors may play an important therapeutic role in patients with GC ineffectiveness or relapse. Lorenzo D et al. reported remission in recurrent type 2 AIP–UC patient after treatment with anti-TNF (adalimumab) (75), which may be related to the fact that anti-TNF therapy can target and block TNF-α, reducing the development of inflammatory processes and activation of immune system cells (119). Recently Lauri G reported the use of ustekinumab for induction and maintenance therapy in type 2 AIP–UC patients with good results (120). Ustekinumab is a monoclonal antibody targeting the p40 subunit of IL 12 and IL 23 (IL12/23p40), IL-23 promotes differentiation of Th cells into Th17 and secretes inflammatory cytokines such as IL-17 and IL-22, whereas IL-12 induces Th1 and produces other cytokines such as interferon-gamma and TNF (121), thus ustekinumab could play an important role in the treatment of type 2 AIP-UC. Neutrophil infiltration is a typical feature of type 2 AIP and UC, and Chiabrando F reported a case report on the use of colchicine, which targets neutrophils, in the treatment of type 2 AIP (122), but colchicine needs to be further explored in the treatment of UC. The neutrophilic capacity of IL-8 makes it a possible link to the pathogenesis of type 2 AIP–UC (68), but anti-inflammatory drugs targeting IL-8 are still being developed (123), which may be an important research direction for the future treatment of type 2 AIP–UC.

At present, there are still some challenges in the diagnosis and treatment of type 2 AIP–UC. On one hand, the diagnosis of type 2 AIP–UC, especially the definitive diagnosis of type 2 AIP, is still difficult (124, 125), and it is necessary to improve the understanding and differentiation of type 2 AIP. On the other hand, the presence of type 2 AIP in UC may indicate a more severe UC phenotype, which may lead to higher rates of colectomy or surgery (9, 126), and this is a challenge for the treatment of type 2 AIP–UC.

## 7 Conclusion

In summary, although type 2 AIP and UC have related features, their comorbidity can affect the clinical presentation of patients and the course of the disease. However, clinical studies on large samples of type 2 AIP–UC are still lacking, as well as basic research on the mechanisms involved. There is still a need to further elucidate the relationship between these two diseases and determine the exact molecular mechanisms. A better understanding of its pathogenesis could help improve disease management and identify potential biomarkers or therapeutic targets.

## Author contributions

NN: Writing – original draft. DW: Writing – review & editing.
